# Lisbon Intensive Falls Trampoline Training (LIFTT) Program for people with Parkinson’s for balance, gait, and falls: study protocol for a randomized controlled trial

**DOI:** 10.1186/s13063-023-07131-4

**Published:** 2023-02-08

**Authors:** Josefa Domingos, John Dean, Júlio B. Fernandes, Catarina Ramos, Miguel Grunho, Luís Proença, João R. Vaz, Catarina Godinho

**Affiliations:** 1 Grupo de Patologia Médica, Nutrição e Exercício Clínico (PaMNEC) do Centro de Investigação Interdisciplinar Egas Moniz (CiiEM), Monte de Caparica, Portugal; 2 Center of Expertise for Parkinson and Movement Disorders, Department of Neurology, Donders Institute for Brain, Cognition and Behaviour, Radboud University Medical Center, Nijmegen, The Netherlands; 3 Triad Health, Aurora, CO USA; 4 LabPSI-Laboratório de Psicologia Egas Moniz, Centro de Investigação Interdisciplinar Egas Moniz (CiiEM), Monte de Caparica, Portugal; 5 Department of Neurology of Hospital Garcia de Orta, Almada, Portugal; 6 Centro de Investigação Interdisciplinar Egas Moniz (CiiEM), Monte de Caparica, Portugal; 7 Egas Moniz Physiotherapy Clinic and Research Centre, Almada, Portugal; 8 CIPER, Neuromuscular Research Lab, Faculty of Human Kinetics, University of Lisbon, Lisbon, Portugal

**Keywords:** Falls, Trampoline training, Effectiveness, Balance, Parkinson’s disease, Randomized controlled trial

## Abstract

**Background:**

Falling and gait difficulties in people with Parkinson’s disease (PD) are associated with impaired reactive postural adjustments and impairments in attentional resources. Combined intensive balance motor and cognitive exercise can be beneficial. However, uncertainties persist regarding the true effects and safety when applying such training. Using trampoline beds may be a favorable safe environment for a highly intensive, cognitive, and balancing training approach. The primary goal of this randomized controlled trial is to assess the effects of an intensive cognitive-motor training program in a safe trampoline environment in addition to usual care on balance impairment, gait, physical capacity, fear of falling, falls frequency and severity, cognition, and clinical impairments in people with mild or moderate PD.

**Methods:**

Sixty participants diagnosed with idiopathic PD, in stage 2–4 Hoehn and Yahr, with a clinical history of gait deficits and a fall history (at least one fall in the last 6 months) will be recruited and randomly allocated to an intervention group receiving cognitive-motor trampoline training or a control group undergoing their usual care. The intervention will consist of 8-week individual training sessions (1-h training, 3 days per week) led by specialized physiotherapists that will provide progressive, challenging training, and guarantee safety. Assessment will be conducted prior to and immediately after the 8-week intervention and at 3 months follow-up after participating in the study. Primary outcome measures will be balance performance (assessed using the Mini-BEST Test and nonlinear analysis) and change in gait parameters (Motor and Cognitive Timed-Up-Go and nonlinear analysis). Secondary outcomes will be change in clinical improvement (Movement Disorder Society Unified Parkinson’s Disease Rating Scale), falls (falls weekly registry), fear of falling (assessed using the Falls Efficacy Scale), physical capacity (6-min walk test), and cognition (Montreal Cognitive Assessment).

**Discussion:**

This study will provide new evidence on the benefits of intensive cognitive-motor balance training on a trampoline for people living with PD. Better guidance on how professionals can apply safer dual-task balance and gait training in rehabilitation is needed.

**Trial registration:**

ISRCTN Registry ISRCTN13160409. Retrospectively registered on February 23, 2022

## Contributions to the literature

- Frequent falls have a significant impact on the mobility and quality of life of people with Parkinson’s disease.
- Supervised exercise programs that challenge balance and also impose cognitive demands have been recommended to improve postural instability and fear of falling, prevent falls, and maintain mobility in people with Parkinson’s.
- This study represents an innovative method to manage balance and falls in Parkinson’s using trampolines to train impaired reactive postural adjustments and impairments in attentional resources in a safe environment. It represents a new guidance on how professionals can apply safer dual-task balance and gait training in rehabilitation.

## Background

Parkinson’s disease (PD) presents many challenges due to the significant functional disabilities affecting posture, gait, daily living activities, and cognition [1]. Falls have a significant impact on the mobility and quality of life of people with PD [2, 3]. They result from a complex interplay of several generic (age-related) and PD-specific fall risk factors [4]. The most frequently highlighted factors are fall history, cognitive impairment, dual tasking, disease severity, postural instability, axial rigidity, shuffling, and small-scaled gait. Frequent falling in people with PD has been highly associated with impaired reactive postural adjustments and impairments in attentional resources [5–7]. When people with PD have to divide attention between two tasks, there can be a decrease in one or both of these tasks. To ensure an appropriate performance, they typically prioritize the cognitive domain, leading to the risk of losing balance and falling due to the lack of sufficient attentional resources [5].

Supervised exercise programs that challenge balance and also impose cognitive demands have been recommended to improve postural instability and fear of falling, prevent falls, and maintain mobility in people with PD [4, 8–10].

Importantly, the combination of both cognitive and motor challenges has been shown to benefit people with PD in both physical and cognitive outcomes [11–15]. Initial research in this area concluded that 30 min once a week for 3 weeks of multiple-task gait training can be feasible in people with PD, with sustained benefits upon multiple-task walking velocity, levels of fatigue, and anxiety [11]. Later, more evidence emerged showing dual-task training can have benefits related to gait (gait speed, step length, and cadence) and balance [9, 16–18].

Even though research has shown the feasibility and safety of dual-task training [18, 19], uncertainties still exist regarding the best way to apply such motor-cognitive exercises that guarantee the highest safety of participants. In clinical practice, physiotherapists and trainers may be reluctant to add dual-task approaches because of safety issues and potential fall risks. Dual-task training was initially recommended not to be used [20] mainly because it was thought to aggravate gait disturbances and cause falls. For example, Conradsson et al. [9] reported 13 falls and an incidence rate of 0.9% during training when applying a high-intensity dual-task walking program in people with PD. Even though the authors reported that none of these events caused injury or pain that interfered with the participants’ ability to proceed with the balance training, safety continues to be a major concern when applying such programs. Given the limited evidence, such programs will thus require specific knowledge and expertise in PD to avoid placing patients at risk and/or receiving unnecessary procedures [21].

The use of trampolines could be one way to guarantee maximum safety while also intensively challenging weight shifting, dynamic changes in balance, aerobic training, strength, and dual-task interference and reduce the risk of injury if a fall should happen. Even though trampoline beds are unstable surfaces, they are expected to guarantee safety while allowing for freedom of movement and training, mimicking better real-life situations. Its use in rehabilitation has been shown to be feasible and efficient in different populations such as older adults [22], individuals with intellectual disabilities [3], and people who suffered a stroke [23]. The study by Aragão et al. [22] showed that a 14-week training on a trampoline improved the ability of older adults to recover balance during forward falls due to a higher rate of hip moment generation. A 2-week trampoline exercise program was an effective intervention to improve the motor and balance ability of school-aged children with intellectual disabilities [24]. Hahn et al. [23] also showed that both the trampoline and the control group had significant improvements in balance, gait, and falls self-efficacy compared to before the 6-week intervention (30 min sessions, 3 times a week) after stroke. In PD, we identified one study that compared the effect of an 8-week rebound therapy-based exercise program over range of motion, proprioception, and quality of life in 20 people with PD [25]. The combination of dual-task training and trampolines has not yet been reported apart from our preliminary results in a pilot study [26]. The results from our group showed that training on a trampoline bed combining balance and cognition was well received and safe for 13 people with PD [26]. Participants reported being “very satisfied” (7/13) or “satisfied” (6/13) with the program. Adverse effects were mild (“feeling tired,” excessive sweating). Participants had favorable perceived benefits (80% very useful; 20% moderately useful), and all indicated that they were willing to continue in the program and recommend it to others.

Considering the preliminary findings regarding compliance, willingness to participate, and safety, here, we aim to assess this program’s effectiveness on clinical outcomes. The primary aim of this proposed randomized controlled trial will be to assess the effects of an intensive cognitive-motor training program in a safe trampoline environment in addition to usual care on balance impairment, gait, fear of falling, falls frequency, physical capacity, cognition, and clinical impairments in people with mild or moderate PD.

## Methods

### Design

The study is a prospective, randomized controlled clinical trial, with a 3-month follow-up, with an allocation ratio of 1:1 and hypothesis type of superiority.

### Study setting

The study will be conducted in collaboration with the Portuguese Parkinson Patient Association (APDPk) and the Movement Disorders Outpatient Clinic from Hospital Garcia de Orta (MDOC-HGO) in Portugal. The program will be delivered in a community environment (trampoline fun house JumpYard) in Lisbon.

### Participants

People with PD will be recruited from the APDPk and the MDOC-HGO by the senior physiotherapist specialist (JD) and the neurologist (MG), respectively. Announcements in common social media channels (Facebook Parkinson’s groups) will also be used to recruit participants and assessed them with their respective neurologists.

Participants will be asked to sign an informed consent and understand their right to withdraw his/her consent at any time without prejudice to future medical care. Participants will be included if (a) diagnosis of idiopathic Parkinson’s disease (Movement Disorder Society Parkinson’s Disease criteria), (b) Hoehn and Yahr stages 2–4, (c) age above 18, (d) able to walk independently and currently able to tolerate a minimum 1 h of exercise, and (e) able to communicate with the investigator, to understand and comply with the study procedures.

Participants will be excluded if they have severe postural instability assessed by MDS-UDPRS Part III item 3.12 and/or severe cognitive difficulties or significant active psychiatric problems that enable the person to participate in the protocol.

### Sample size calculation

The sample size for this study was determined based on previous related interventional trials with people with PD. The sample size was determined considering a two-sided alpha level and was based on a difference between two independent group means (*T*-Student’s test). Considering a 0.7 effect size (e.g., for the FES-I outcome), for 80% power and an alpha level of 5%, a sample size of 26 subjects per group will be required. With a dropout rate set at 15%, a final sample of 60 (*n* = 30 per group) was considered optimal. So, we will include 60 people with PD living in Portugal.

### Randomization and blinding

**Fig. 1:**
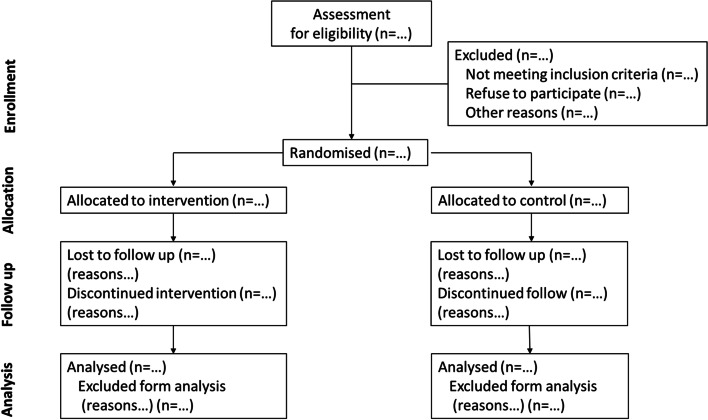
Flowchart describing the search strategy

After baseline assessments (T0), participants will be divided into two groups (one experimental group with trampoline training [EG] and one control group [CG]). Online software will be used to generate the randomization plan using a random block sizes method. This randomization procedure assures an allocation concealment format—i.e., the individual in charge of this task does not know what the next group allocation will be. This researcher will assign participants to their groups and perform follow-up measurements (Fig. 1). The data analysts will be blind to the group control or intervention.

### Interventions

The intervention for the EG will consist of an 8-week individual training regime (1-h individual training, three times per week). Sessions will be led by a physiotherapist specializing in PD and motor and cognitive exercise programs. This health professional will ensure adaptation, specificity, progression, variation, and safety of the training. Guidance for exercise in PD was underpinned by clinical experience and evidence from the European Physiotherapy Guidelines for PD [20]. Each session will start with a 10-min warm-up outside of the trampolines, comprising a variety of whole-body amplitude-based movements and walking activities. It will end with a 10-min cooldown incorporating breathing and low amplitude movements as active stretching. The sessions will consist of performing motor exercises on the trampolines while adding in motor and cognitive exercises that will progressively be increased. Motor exercises will include an array of frequently recommended movements for PD that directly enhance specific functional activities of daily living, such as multidirectional stepping in place, sitting and standing, standing with good posture under unexpected balance challenges, reaching in diverse directions (e.g., catching a ball), carrying soft objects (while standing and walking), walking, turning and running, and getting up from the floor/trampoline [20]. All these physical exercises will focus on reinforcing the use of high-amplitude, multidirectional movements with increasing speed and complexity. Because people with PD often depend on cues when performing motor tasks during the sessions, cueing strategies (cognitive, visual, tactile, or verbal) will be used according to the patient’s needs to promote good-quality movement and reduce compensations [20, 27, 28]. Cognitive exercises will target the 4 main cognitive domains particularly affected by PD: attention, working memory, executive function, and visual exploration [29]. The dual cognitive tasks will include standing, sitting/standing, stepping, or walking while performing cognitive tasks such as simple talking, memorizing lists of words, alternating sentences, spelling words backward, counting a 7-digit number forward/backward, and quick decision-making.

**Fig. 2:**
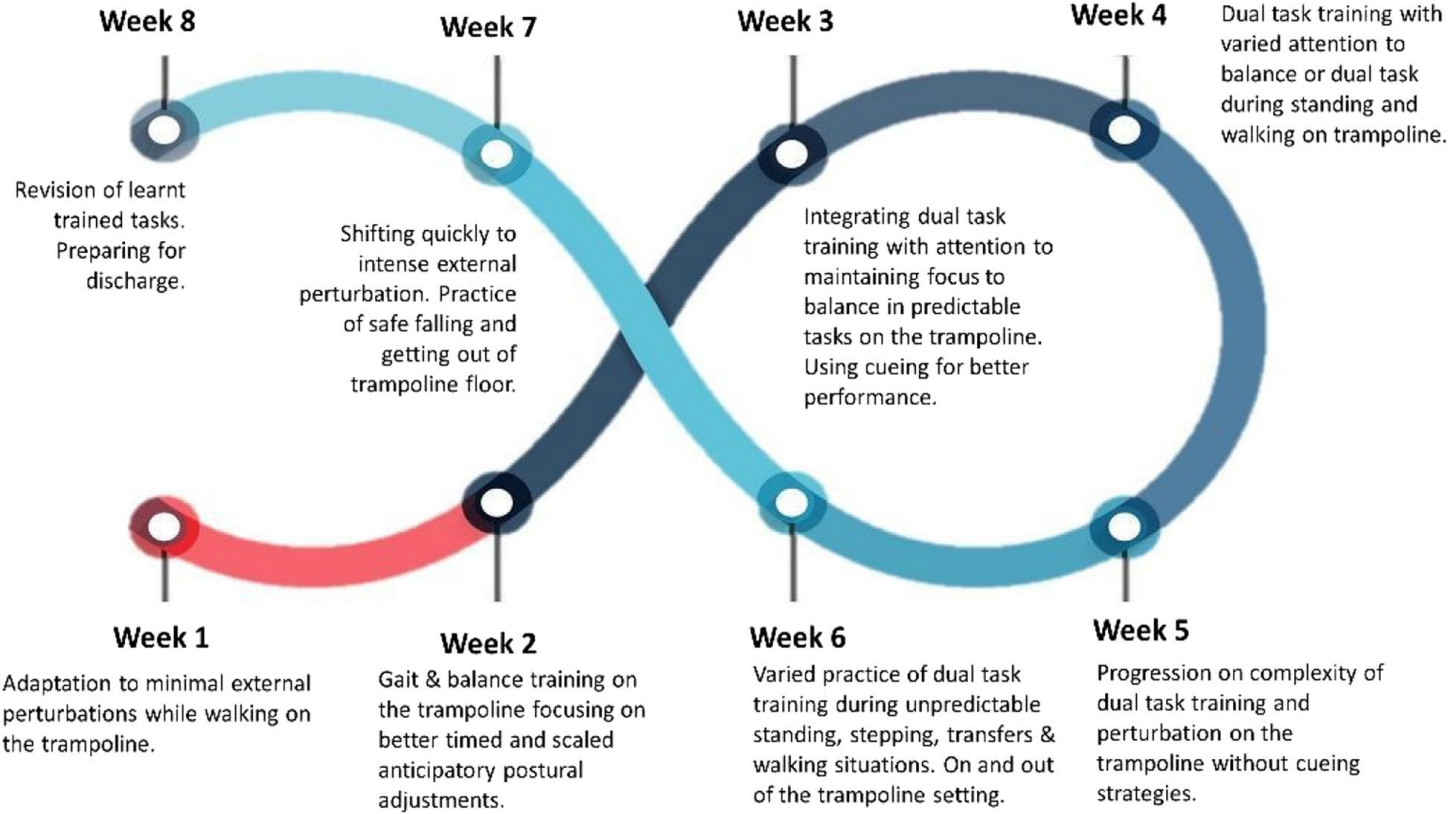
Lisbon Intensive Falls Trampoline Training (LIFTT) Program weekly progression

In Fig. 2, we can see a summary of the progression of activities without a very restrictive protocol to assure its specificity to the patient and also to better transfer to clinical practice. During the sessions, the therapist is required to modify the exercises for the patient and may include adjustments to the type of physical activities, length, use of verbal feedback, time for learning, and number and type of cognitive exercises added. Feedback from each session will be used to adapt the program to the personal needs of each participant. The proposed protocol is not restrictive, aiming to be highly focused on the individual dosage and specificity, which ultimately better represents and has a real impact on clinical practice.

Additionally, safe fall landing strategies will be taught and reinforced throughout the program but specifically trained in the last two weeks. There is emerging evidence to suggest that these landing strategies can be safe and effective for a geriatric population [30]; however, this intervention has not been examined in a clinical physical therapy practice setting in PD. This will be an additional novelty of our program.

At the end of each session, the therapist will document in the study log any adverse event, patient feedback about the exercises and need for support, motives for absence, and any reported falls that occurred during the training.

To ensure participant retention, we facilitate traveling with reimbursement of costs and a list of concierge services to choose from. The team applying the intervention will be asked to express appreciation weekly, do follow-up calls when someone is absent, and share weekly results reached.

In the control group, participants will continue to receive their usual care from the health services and only receive the assessments. The intervention group will go back to usual care during follow-up.

### Data collection

Assessments will take place at APDPk by a blinded researcher in the week before (T0), after 8 weeks of training protocol (T1), and 3 months after the end of the program (T2). The same protocol will be applied, composed of scales/questionnaires and validated assessment instruments, referenced below. The researchers who will perform these assessments will not participate in the intervention. All participants will be assessed in the ON medication phase with the following specific clinical scales and tests that will have their order randomized. Patients will be assessed for (a) balance (Mini-BEST Test) [12], (b) gait (Motor and Cognitive Timed Up Go (TUG)) [12], (c) clinical impairments (Movement Disorder Society Unified Parkinson Disease Rating Scale (MDS-UPDRS)) [31], (d) frequency and severity of falls (falls weekly registry) [12], (e) fear of falling (Falls Efficacy Scale (FES-I)) [32], (f) physical capacity (6-min walking distance test (6MWD)) [12], (g) cognition (Montreal Cognitive Assessment (MoCA)) [33], and (h) quality of life (PDQ-8) [34, 35]. Additional gait and balance assessments will be conducted using accelerometers placed on the ankles and standing on a force plate. Participants will be asked to walk on a treadmill for 10 min at their preferred walking speed. Moreover, their balance will be tested in both eyes opened and closed conditions. They will be asked to remain still for 2 min. Researchers that will conduct the assessments will be trained by a senior and experienced researcher. The database obtained will undergo a double check (two different researchers) to identify potential failures/errors.

### Data analysis

We will perform descriptive and inferential statistics of data collected at T0, T1, and T2, to identify the differences between the two groups (EG and CG) and to evaluate which of these groups will have better results as a function of time (from T0 to T1 and T2). Assessment of reliability and correlation analyses will also be conducted. We do not anticipate the possibility of performing subgroup analysis for this study. The protocol will not change during the entire interventional period of the study; therefore, no interim analysis will be performed, nor do we anticipate the need for stopping guidelines.

For achieving the objective mentioned above, we will use a factorial (two-way) repeated measures (RM) ANOVA, considering a between-subject factor (treatment: EG, CG) and a repeated measures factor (time: T0, T1, T2). The two-way RM ANOVA will allow to evaluate the individual contribution of each factor and their interaction throughout time. The latent growth modeling (LMG) technique will be conducted to establish the effectiveness of the individual intervention on the growth of primary outcomes during the period between the beginning (T0) and the end (T2) of the program.

Regardless of the potential dropouts missing data, we will include all the participants in the statistical analysis (i.e., intention-to-treat analysis).

### Ethics and dissemination

The Lisbon Intensive Falls Trampoline Training (LIFTT) Program includes two major ethical areas such as humans in general and the protection of general personal data issues. Regarding the protection of general personal data, LIFTT will follow all requirements of GPDR with a particular focus on Article 29 Workgroup conclusion on anonymity and informed consent on their questionnaires and collected data. In general, the public impact/interest of LIFTT in Humans will allow the reduction of gaps in science, particularly in PD rehabilitation and in patient’s autonomy which leads to an improvement in patient’s quality of life. In this randomized controlled clinical trial, patients will be assessed and trained for motor skills with noninvasive techniques, and their functional status will be studied using standard metrics for PD.

The informed consent form will contain comprehensive information about contents, objectives, duration, procedures, voluntariness, and possible risks of study participation. It will be emphasized that the participant is at liberty to withdraw their consent to participate at any time without penalty or loss of benefits to which the participant is otherwise entitled. Participants who refuse to give or withdraw written informed consent will not be included or continued in this study, but this will not impact their subsequent care. During the screening visit, the participants will be given an explanation of the objective and compliance needed for the study. Any questions will be considered and answered. In case of an agreement to voluntarily participate in the study, the participant must sign two copies of the informed consent form; one will be given to the participant, and the other form will be retained at the local study center. The consent forms will be kept separately from the data. Essential documents will be archived in a way that ensures that they are readily available, upon request, to the competent authorities. Case report forms (CRFs) will be used as source documents for collecting data. These CRFs will be encoded (a coded combination of numbers and letters, e.g., LIFTT 01), so that it is impossible to assign the data contained in a CRF to an individual participant. Only the project coordinator will have access to the decoder grid. All paper copies will be stored in a locked file, as well as all electronic data. Data will be maintained for 5 years in a restricted access locked file cabinet at Egas Moniz - Cooperativa de Ensino Superior, C.R.L. After that, they will be destroyed. All data will be confidentially locked, and all subject information will remain anonymous.

Participants who are unable to continue to participate in the intervention will withdraw from the intervention. On withdrawal, we will register the date and reason for withdrawal.

They will also continue to be followed up and data collected until the end of the study unless they explicitly withdraw their consent.

Regarding drafting, editing, accountability of the data, and study authorship, we will follow the current version of the International Committee of International Journals Editors (ICMJE) recommendations. The data obtained will only be used in the context of this study and professional writers will not be hired for this study.

All items from the World Health Organization Trial Registration Data Set are found within the protocol. The project has been approved by the Ethics Committee of Egas Moniz - Cooperativa de Ensino Superior, C.R.L., ref: 1052/2022 on January 27, 2022.

## Discussion

This randomized controlled trial will assess the benefits of an intensive cognitive-motor balance and gait program for people with PD undertaken in a safe trampoline environment. We expect that training on a trampoline surface will challenge the balance system while giving a sense of safety and will be effective for balance outcomes, showing an increase in the Mini-BESTest score. Providing care in community settings may not only enhance participation in the study but also show how exercise programs can easily be incorporated into the normal routine and structure of a community setting.

While addressing as many fall risk factors as possible in PD is critical, whether that be addressing medications to reduce OFF freezing or making alterations to improve home safety, the importance of combining balance and cognitive training cannot be overstated. Because gait and balance deficits in PD are aggravated under dual or multitask conditions, effective and safe training to enhance dual-task performance in standing and walking is critical for PD rehabilitation. This study represents an innovative combined approach to train balance, gait, and falls in real-life safe community settings in PD. If proven effective, our results should benefit people with PD by safely improving their balance and gait patterns. Additional benefits over clinical disease management, cognition, physical capacity, and fear of falling are also expected. This will ultimately promote the safe, effective, and appropriate use of nonpharmacological interventions such as physiotherapy in worldwide health care systems.

Using new training approaches such as exercises on a trampoline can help health professionals to better train motor and cognitive performance while guaranteeing safety and falling without injury. Importantly, using large-sized trampoline beds (a differentiating factor) allows participants to train balance exercises more freely and mimic challenges that are more like real-life situations where people are challenged to keep their balance under dual-task conditions. More significant transfer effects will occur in the conditions that are trained, so the closer the activity is to how it happens in real life, the greater our chances of improving it outside the clinic [20]. Additionally, just challenging balance training has been shown to improve gait in mildly to moderately affected patients in programs without gait training [36].

The intensity of the training plays an important role in expected outcomes [9, 37, 38]. Previous research showed that a high balance intensity dual-task walking program benefited balance and gait abilities in people with PD performed when applied in groups of four to seven participants, three times per week, 60 min per session, for 10 weeks at a university hospital [9]. Notably, later, a 6-week home program (one session per week with the therapist for six consecutive weeks and a weekly self-directed home program) that combined strength, movement strategy training, and falls education did not prevent falls in the long term due to insufficient dosage of therapy delivered [38].

Additionally, identifying effective outcome measures that are responsive to such interventions is also needed to improve the ability to design future clinical studies with such programs. The results will also contribute to the urgent knowledge needed to define clear dual motor-cognitive task recommendations for PD.

We expect one limitation to the proposed study regarding the lack of an active control group. In Portugal, there is no uniform standard physiotherapy treatment for people with PD. For this reason, this study will compare its results to the effect of physiotherapy treatment considered usual for each individual patient (no physiotherapy, falls education, exercise in the community, number of sessions of rehabilitation).

## Conclusion

There is a clear need to implement care that reduces the pitfalls in managing the frequency and severity of falls in PD for healthcare professionals, healthcare systems, and all of society. For healthcare professionals, care delivery in a salient, safe, and progressively complex training mode is a promising and exciting step forward in managing balance and gait deficits in PD while keeping patients engaged in ongoing treatments. For healthcare systems, trampoline exercises with motor-cognitive challenges in PD represent a practical and cost-effective training mode with a significant impact on balance, fear of falling, frequency and severity of falls, gait, and overall improvements in cognitive function. It will also allow us to apply a novel approach to teach older adults with PD the movement strategies to fall safely.

At a research level, this randomized controlled trial can stimulate other groups to study similar protocols and provide new knowledge in the fall arena and a paradigm shift in balance and gait interventions in PD.

## Trial status

Trial registration: ISRCTN13160409. Lisbon Intensive falls Trampoline Training for Parkinson’s: The LIFTT Program. Retrospectively February 23, 2022.

This study is in the recruitment phase with the completion of recruitment scheduled for February 2023.

Date and version identifier: January 2023 protocol version 1.

## Data Availability

The datasets used and analyzed during the current study are available from the corresponding author upon reasonable request.

## Abbreviations

APDPk: Portuguese Parkinson Patient Association
CRFs: Case report forms
LIFTT: Lisbon Intensive Falls Trampoline Training
MDOC-HGO: Movement Disorders Outpatient Clinic from Hospital Garcia de Orta
PD: Parkinson’s disease
